# The Neuro Patterns Prior to Error Responses in Long-Lasting Working Memory Task: An Event-Related Potential Study

**DOI:** 10.3389/fnbeh.2019.00277

**Published:** 2019-12-18

**Authors:** Yi Xiao, Jintao Wu, Jiaxuan Li, Weicai Tang, Feng Ma, Chenhui Sun, Yuan Yang, Wenhao Zhan, Lizhi Wang, Huijong Yan, Fenggang Xu, Shanguang Chen

**Affiliations:** ^1^National Key Laboratory of Human Factors Engineering, China Astronaut Research and Training Center, Beijing, China; ^2^School of Biological Science and Medical Engineering, Beihang University (BUAA), Beijing, China; ^3^School of Aerospace, Tsinghua University, Beijing, China; ^4^Cadre Ward Section, 306 Hospital of PLA, Beijing, China

**Keywords:** error, neural pattern, fatigue, ERP, working memory

## Abstract

Few studies exist regarding the mechanism prior to response by which cognitive impairment may induce error in a single long-lasting task. The present study intends to clarify the changes in cognition at the electrophysiological level. Changes in amplitude and latency of N1, P2, N2, and P3 components of event-related potentials (ERPs) were analyzed for error and correct trials during normal and fatigue. Twenty-nine participants had to perform a 2-back working memory (WM) task for 100 min. The first 10 min and the last 10 min of the task were used as the normal state and fatigue state of the participant, respectively. EEG data were obtained from the first 10-min period and the final 10-min period. The results revealed smaller P3 and P2 amplitudes and longer P2 and N2 latency in the final 10-min which was after a long-lasting time task. Moreover, smaller P3 and P2 amplitudes but larger N2 amplitudes were observed in error trials for both states. Our results indicated that: (1) long lasting involvement in a cognitive task had a detrimental effect on attention, memory updating and cognitive control; and (2) impaired attention, impairments in memory updating and cognitive control were related to task errors. Our results imply that several impaired cognitive processes were consistently associated with the error and the altered ERP represents the neural patterns prior to error response in mental fatigue state.

## Introduction

Long-lasting engage in a cognitive task leads to impair attention, working memory (WM) and cognitive control. This phenomenon is named mental fatigue (van der Linden et al., 2003; Boksem et al., 2005, 2006; Boksem and Tops, 2008; DeLuca et al., 2009; Kato et al., 2009; Marcora et al., 2009; Tanaka et al., 2012; Möckel et al., 2015; Rozand et al., 2015; Xiao et al., 2015; Smith et al., 2016). It has been demonstrated that mental fatigue led to increase errors and even might induce accidents (Swaen et al., 2003; Noy et al., 2011). There have been many studies showing impaired cognitive ability in mental fatigue. However, the relationship between the impaired cognition and error in mental fatigue is still unknown.

N1, N2, P2, and P3 are the event-related potentials (ERPs) components that reflect different functions in many cognitive processes (Luck, 2005), which are affected by mental fatigue (van der Linden et al., 2003; Boksem et al., 2005, 2006; Royal et al., 2006; Cook et al., 2007; Lorist, 2008; Wright et al., 2008; Kato et al., 2009; Langner et al., 2010; Duncan et al., 2015; Rozand et al., 2015; Smith et al., 2016). N1 is sensitive to the physical features of stimuli and reflects the recognition and coding processes (Taylor, 2002; Morgan et al., 2008). The N1 amplitude was decreased in mental fatigue (Boksem et al., 2005; Möckel et al., 2015) N2 is closely related to cognitive control in memory (Daffner et al., 2011a; Gajewski and Falkenstein, 2014). Some studies (Boksem et al., 2005; Möckel et al., 2015) reported N2 amplitude increased after a long-lasting task. Furthermore, some studies in older and patients also found that increased N2 amplitude reflected an enhanced energetic cost in cognitive control (Bruder et al., 1998, 2001; Nieuwenhuis et al., 2005; Daurignac et al., 2006; Guillem et al., 2006; Folstein and Van Petten, 2008; Daffner et al., 2011b; O’Connell et al., 2012; Shu et al., 2014; Sumich et al., 2014; Pinal et al., 2015a; Zuj et al., 2017), suggesting that the patients used more resources for the response selection. These results are consistent with the mental fatigue effect. P2 is part of memory information process related to the onset of memory updating (Taylor et al., 1990; Lefebvre et al., 2005; Evans and Federmeier, 2007; Lenartowicz et al., 2010). One study (Lorist, 2008) found that mental fatigue diminished P2 amplitude. P3 is regarded as a very important ERP component related to attention, cognitive resource reallocation and memory updating. The P3 amplitude was decreased in no-go trials but remained unchanged in Go trials; in both cases, latencies were longer during mental fatigue (Kato et al., 2009). The results showed that resource allocation was attenuated in mental fatigue for no-go stimuli.

However, few studies exist to examine which cognitive processes are impaired and thus are responsible for inducing increased errors in a single task under mental fatigue. Previous studies regarding the reasons for task errors in normal state give us a starting point (Ridderinkhof et al., 2003; Hajcak et al., 2005; Padilla et al., 2006; Maidhof et al., 2009; Ruiz et al., 2009; Masaki et al., 2012; Wessel et al., 2012; Bode and Stahl, 2014; Ora et al., 2015; Shou et al., 2015). Some studies have also suggested that errors increase when many cognitive processes are impaired simultaneously (Padilla et al., 2006; Mathewson et al., 2009; Mazaheri et al., 2009; Eichele et al., 2010; Bode and Stahl, 2014; Ora et al., 2015; Shou et al., 2015; Xiao et al., 2019).

In nature, when cognitive processes are impaired, the brain cannot supply sufficient cognitive resources for perception or response, leading to an error in some trials. Therefore, we believe that a higher error rate in the mental fatigue state can be attributed to a decline in the functioning of cognitive processes. Furthermore, a process with higher cognitive demand, especially, the target-driven attention system was easily affected in fatigue (Boksem et al., 2005, 2006). The mechanisms underlying error during WM tasks in a fatigued state have yet to be elucidated (Chen et al., 2008; Lenartowicz et al., 2010; Xiao et al., 2019). With respect to the 2-back WM task used in this study, the stimuli selected are very easy to recognize, and the stimulus recognition is not easily impaired. Therefore, there may be no difference in N1 between the normal and fatigue states. However, the cognitive demand for updating and response selection processes is increased, so the cognitive functions should be easily affected in fatigue (Boksem et al., 2005, 2006). Therefore, the amplitudes of P2 and P3 may decrease in the fatigue state. The N2 amplitude should increase in fatigue based on the previous studies (Daurignac et al., 2006; Guillem et al., 2006; Daffner et al., 2011b; O’Connell et al., 2012; Shu et al., 2014; Sumich et al., 2014; Pinal et al., 2015a; Zuj et al., 2017). Our previous work (Xiao et al., 2019) was the first study on the neuro mechanism of errors and it showed that a deficit in the ability of memory updating and decreased cognitive control causing an error. So, the P2 and P3 amplitudes would decrease in error trials, and the N2 amplitude in error trials would increase. Hence, our hypothesis is that the mental fatigue impairs the memory updating and cognitive control which induce more error. In all, the P2, P3 and N2 would change in the mental fatigue and in error trials as well. And more errors would be attributed to the P2, P3 and N2 change in mental fatigue state. Therefore, we analyzed the differences in N1, P2, N2 and P3 between normal and fatigue states and between correct and error trials.

## Materials and Methods

### Participants

A total of 36 right-handed male adult individuals (mean age = 25 ± 2.91 years; education = postgraduate or graduate students from BeiHang University, China) participated in the study. All participants had a normal or corrected-to-normal vision and did not have any psychiatric disorders. The informed written consent and course requirements were provided. The whole study was approved by the committee of the China Astronaut Research and Training Center. The ethical guidelines for the experiment were in accordance with the Declaration of Helsinki. Seven participants were excluded for excessive artifacts. Finally, the remaining 29 subjects who had more than 20 errors were chosen for further analysis.

### 2-Back Task

**Figure 1:**
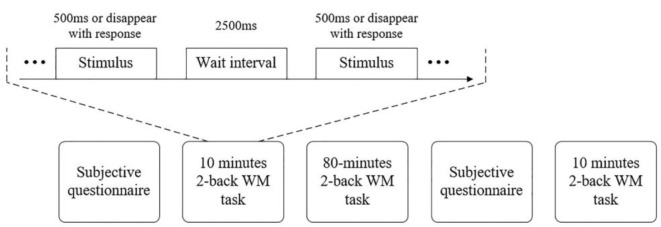
The procedure.

The experimental task is summarized in Figure 1 and was presented using E-Prime software. The pseudorandom digits 1, 2, 3, 4, 5, 6, 7, 8, and 9 were displayed individually in white against a black background on a 24-inch LCD screen with a refresh rate of 60 Hz. The stimuli measured 1.8 cm high by 1.4 cm wide. The distance between the LCD screen and the participants was 60 cm to 80 cm. Each stimulus was displayed for 500 ms or disappeared earlier if a response was recorded before the time elapsed. After 2,500 ms, a new stimulus was displayed. Participates were asked to press the “F” or “J” button as accurately and quickly as possible. They were required to determine if the presented stimulus matched the one presented two trials before. If it matched, they had to press the “F” button. In contrast, if it did not match, they needed to press the “J” button. The rate for the target was 33.33%. The number of trials was probably due to the reaction times (RTs), so the number was different between participants. However, from the task design the number was 200 at least.

### Procedure

Recruitment materials provided a general description of the study but did not reveal its purpose. Each participant was asked to attend a training before the formal experiment (i.e., 1 day before) to ensure that all subjects would be familiar with the tasks so as to eliminate learning effects in a formal experiment. The 100-min 2-back WM task was segmented into three subtasks, like the experiment in the literature (Möckel et al., 2015), the long-lasting task of which was divided into three equal blocks, and it induced fatigue. The first 10 min was the normal state subtask, and the last 10 min was the fatigue state sub–task. The 80-min 2-back WM task in between the normal and fatigue subtasks was used to induce fatigue. The questionnaires were recorded before the start of the task and just before the last 10 min.

### Fatigue Questionnaire Data

The questionnaire (Yoshitake, 1971; Xiao et al., 2015) was a five-item self-reporting inventory that was designed to measure transient or fluctuating affective states. The five items were mental clarity, attention concentration, sleepiness, comprehensive assessment of fatigue (i.e., the fatigue subscale) and emotion. The participants were required to describe and score their feelings on a scale that ranged from 1 (least fatigue) to 10 (extreme fatigue).

### EEG Recording and Analyses

The EEG and electrooculography (EOG) data were recorded by 63 sintered Ag-AgCl electrodes mounted in an elastic cap (EasyCap, Brain Products GmbH) with a standard 10/20 system layout. The reference electrode was FCz and the ground electrode was AFz. The impedance was kept below 5 kΩ. Signals were recorded by the Brain Vision Recorder (Brain Products GmbH, Ver. 1.03) with 1,000 Hz sampling rate and bandpass filtered at 0.01–250 Hz. The signals were amplified in the range of ± 3.27 mV and at a resolution of 0.1 μV. The horizontal EOG was recorded at the outer corner of the eyes and vertical EOG at the upper and lower corner of the right eye.

The EEG data were processed by the Brain Vision Analyzer 2.0. Software (Brain Products GmbH, Germany). Tp9 and Tp10 were chosen as the new references (Brain Products GmbH; Möckel et al., 2015). The raw data were inspected using the semi-automatic inspection method. The gradient criterion was 50 μV/ms. The allowed maximum absolute difference was 200 μV with a 200-ms interval. The maximum amplitude was −200 μV to 200 μV. The lowest amplitude was 0.5 μV. After raw data inspection, the eye movement artifacts were corrected based on Gratton and Coles’ algorithm. The EEG were filtered offline using a 2nd order Butterworth filter in the 0.1–35 Hz range with 0-phase shift 48 dB, and the notch filter was 50 Hz. Stimulus-locked data were segmented into epochs of −200 ms to 800 ms after stimulus presentation, and the 200-ms interval before stimulus presentation was set as baseline. Accepted trials were averaged separately for correct responses and errors. Maximum amplitudes of N1, N2 and P3 were measured at Fz, Cz and Pz from intervals of 130–170 ms, 270–360 ms, 400–500 ms poststimulus. P2 was measured as the maximum amplitudes at Fz and Cz from intervals of 170–270 ms, post-stimulus. For each ERP component, the average of the 10 points around the peak amplitude were considered the maximum amplitudes.

### Statistical Analysis

The five subscales were calculated for normal state and fatigue state. In addition, the paired *t*-test was used to analyze the two different states (Hsieh et al., 2010; Murphy et al., 2010). Descriptive statistics of error rate, RTs of error and correct trials, and the total number of errors in fatigue and normal states were computed. The error rate is defined as the number of error keystrokes divided by the total number of keystrokes. Error and omission rates were examined for fatigue and normal states by the paired *t*-test (Hsieh et al., 2010; Murphy et al., 2010). The RTs of error and correct trials were examined by repeated-measures ANOVA with the state (fatigue vs. normal, 2), trial type (correct vs. error, 2). All component amplitudes except P2 were analyzed by two-way repeated-measures ANOVA with the state (fatigue and normal, 2), trial type (correct and error, 2) and electrode (Fz, Cz, and Pz, 3) as within-subjects factors. The P2 amplitudes were analyzed by two-way repeated-measures ANOVA with the state (fatigue vs. normal, 2), trial type (correct and error, 2) and site (Fz and Cz, 2) as within-subjects factors. Latencies were analyzed by two-way repeated-measures ANOVA with state (fatigue and normal, 2) and trial type (correct and error, 2) as within-subjects factors (Gehring and Fencsik, 2001).

## Results

### Questionnaire Data

**Figure 2:**
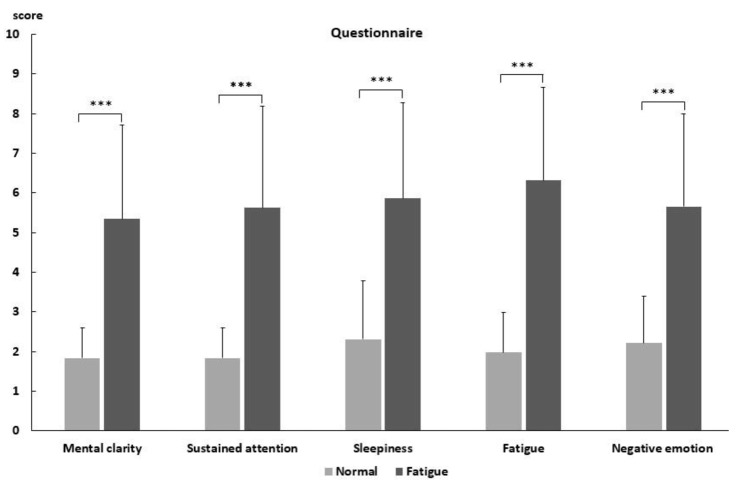
The questionnaire results for normal and fatigued state. ***Difference is significant at the 0.01 level.

As shown in Figure 2, feelings of mental fatigue increased while participants finished the 80 min task. The scores on the sleepiness subscale (*t* = −6.192, *p* < 0.001) and the fatigue subscale (*t* = −8.831, *p* < 0.001) were much higher after the 80-min task. Decreases in mental clarity (*t* = −7.942, *p* < 0.001) and sustained attention (*t* = −7.909, *p* < 0.001) and an increase in negative emotion (*t* = −7.26, *p* < 0.001) were observed as well.

The results showed that mental fatigue led to negative affection on the participants. The five-item self-reporting inventory showed that much more negative affective states after long-lasting task.

### Behavioral Results

**Figure 3:**
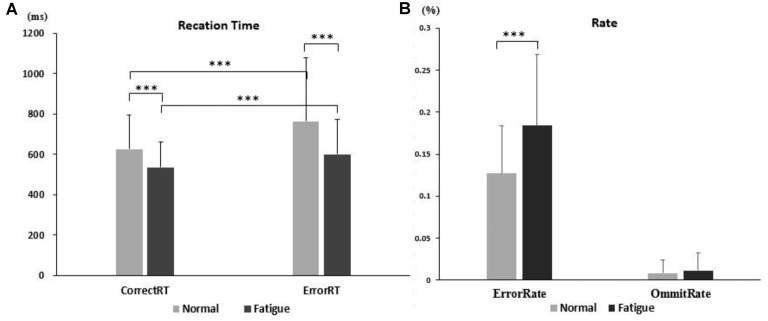
The behavioral results. **(A)** The results of reaction times (RT). **(B)** The results of error rate and omission rate. ***Difference is significant at the 0.01 level.

As shown in Figure 3, the error rate increased (0.184 ± 0.085 vs. 0.1268 ± 0.0572; *t*_(28)_ = −3.181, *p* = 0.004), but the omission rate (0.011 ± 0.022 vs. 0.008 ± 0.016; *t*_(28)_ = −0.832, *p* = 0.412) did not change in fatigue. The RTs for error trials (600.597 ± 173.869 vs. 765.451 ± 313.708; *F*_(1,28)_ = 9.008, *p* = 0.006, $\eta_{\text{p}}^{2}$ = 0.243) and for correct trials (537.456 ± 124.441 vs. 626.451 ± 167.168; *F*_(1,28)_ = 8.783, *p* = 0.006, $\eta_{\text{p}}^{2}$ = 0.239) were significantly shorter in the fatigue state.

For trial type, the RTs for correct trials (626.451 ± 167.168 vs. 765.451 ± 313.708; *F*_(1,28)_ = 17.378, *p* = 0.000 $\eta_{\text{p}}^{2}$ = 0.383) were significantly shorter than RTs for error trials in the normal state. In the fatigue state, the RTs of correct (537.456 ± 124.441 vs. 600.597 ± 173.869; *F*_(1,28)_ = 13.066, *p* = 0.001, $\eta_{\text{p}}^{2}$ = 0.318) were still significantly shorter than RTs of error trials.

The RTs for correct trials were significantly shorter than RTs for error trials. Moreover, the RTs were shorter in mental fatigue (a). The results showed that mental fatigue led to more errors (b).

### ERPs Results

**Figure 4:**
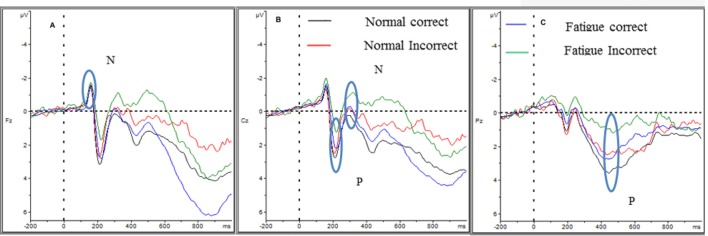
ERPs of correct and error response at normal and fatigue. Panels **(A–C)** show the N1, P2, N2, and P3of the two trials in two-state. Panel **(A)** shows the four event-related potentials (ERPs) in Fz; panel **(B)** shows the four ERPs in Cz; and panel **(C)** shows the four RRPs in Pz. The N1 amplitude showed no difference. The P2 and P3 amplitudes diminished in fatigue state and error trials as well, while the N2 amplitude was larger.

**Figure 5:**
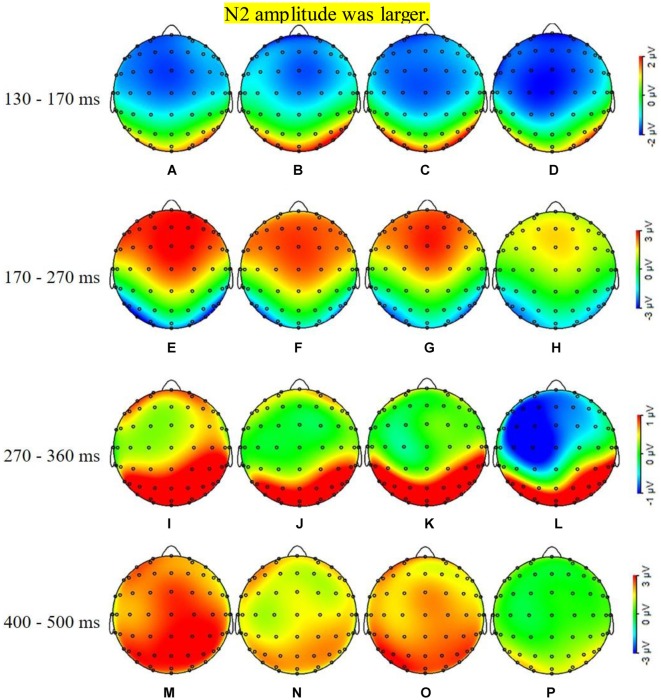
ERPs mapping of correct and error trials at the time interval of peak amplitudes. Panels **(A,B)** show the N1 mapping of correct and error ERP in normal, panels **(C,D)** show the N1 mapping of correct and error in fatigue, respectively; there is no difference at the prefrontal and central areas in the four figures. Panels **(E,F)** show the P2 mapping of correct and error ERP in normal, panels **(G,H)** show the P2 mapping of correct and error in fatigue, respectively; the amplitudes in fatigue state and the amplitudes of error were smaller in the Fz and Cz. Panels **(I,J)** show the N2 mapping of correct and error ERP in the normal state, panels **(K,L)** show the N2 mapping of correct and error in fatigue, respectively; the amplitudes of error were larger in the Fz, Cz, and Pz. Panels **(M,N)** show the P3 mapping of correct and error ERP in the normal state, panels **(O,P)** show the P3 mapping of correct and error in fatigue, respectively; the amplitudes of fatigue and the amplitudes of error were smaller in the Fz, Cz, and Pz.

The mean ERPs amplitudes and latencies are shown in Tables s 1, 2. The ERPs waves and mapping for error and correct trials in normal and fatigue states for all participants are shown in Figures 4, 5.

**Table 1:** Event-related potentials (ERPs) amplitude (μV).

ERPs component	State	Electrode	FZ		Cz		PZ	
		Trial type	Correct	Error	Correct	Error	Correct	Error
N1	Normal	Mean	−1.89	−2.00	−1.85	−1.98	−1.06	−1.27
		Std	1.20	1.42	1.30	1.33	1.10	1.55
	Fatigue	Mean	−1.64	−1.81	−1.86	−2.16	−0.98	−1.54
		Std	1.26	1.61	1.24	1.47	1.04	1.43
N2	Normal	Mean	−0.72	−1.25	−0.75	−1.43	−0.87	−1.19
		Std	2.31	2.96	2.10	2.80	1.84	1.94
	Fatigue	Mean	−0.43	−1.34	−0.63	−1.64	−0.27	−0.88
		Std	2.02	2.10	1.81	1.94	1.71	2.18
P2	Normal	Mean	3.93	3.60	3.55	3.30	/	/
		Std	1.67	1.86	1.75	1.77	/	/
	Fatigue	Mean	3.45	2.56	2.87	2.09	/	/
		Std	1.64	1.66	1.57	1.54	/	/
P3	Normal	Mean	2.10	1.53	2.66	1.83	4.23	3.29
		Std	3.00	3.48	3.21	3.50	2.50	1.87
	Fatigue	Mean	1.64	0.95	2.00	0.68	3.47	2.17
		Std	2.23	2.14	2.19	1.99	2.14	2.16

**Table 2:** ERPs latency (ms).

ERPs	Type	Correct	Error
N1	Normal	156.34 ± 10.92	152.59 ± 12.73
	Fatigue	158.41 ± 9.90	155.03 ± 13.24
N2	Normal	290.10 ± 29.33	290.48 ± 29.39
	Fatigue	298.83 ± 24.70	307.31 ± 29.51
P2	Normal	213.31 ± 20.74	213.52 ± 23.67
	Fatigue	227.79 ± 20.07	219.1 ± 22.53
P3	Normal	290.1 ± 29.33	290.48 ± 29.39
	Fatigue	298.83 ± 24.70	307.31 ± 29.51

For N1 amplitude, there was no main effect of state, *F*_(1,28)_ = 0.02, *p* = 0.961, $\eta_{\text{p}}^{2}$ < 0.0 01. Furthermore, there was no main effect of trial type (correct and error), *F*_(1,28)_ = 3.327, *p* = 0.079, $\eta_{\text{p}}^{2}$=0.106. However, a significant difference between electrodes was revealed, *F*_(1.183,33.125)_ = 7.91, *p* = 0.006, $\eta_{\text{p}}^{2}$ = 0.220. The Cz electrode had the largest amplitude and the Pz electrode had the smallest amplitude. No interaction effects were significant, including the interaction of state * trial type (*F*_(1,28)_ = 0.393, *p* = 0.536, $\eta_{\text{p}}^{2}$ = 0.014), the interaction of state * trial type (*F*_(1.126,31.527)_ = 1.318, *p* = 0.265, $\eta_{\text{p}}^{2}$ = 0.045), the interaction of trial type * electrodes (*F*_(1.655,46.348)_ = 2.1, *p* = 0.142, $\eta_{\text{p}}^{2}$ = 0.07) and the interaction of state * trial type * electrode (*F*_(1.604,44.924)_ = 0.438, *p* = 0.604, $\eta_{\text{p}}^{2}$ = 0.015).

We found no main effect of state on N1 latency *F*_(1,28)_ = 1.708, *p* = 0.202, $\eta_{\text{p}}^{2}$ = 0.057 and no main effect of trial type, *F*_(1,28)_ = 3.789, *p* = 0.062, $\eta_{\text{p}}^{2}$ = 0.119. No interaction effects of state * trial type *F*_(1,28)_ = 0.01, *p* = 0.919, $\eta_{\text{p}}^{2}$ < 0.001 were significant.

For P2 amplitude, there were main effects of state *F*_(1,28)_ = 14.392, *p* < 0.001, $\eta_{\text{p}}^{2}$ = 0.339, and the amplitude was decreased in fatigue. Furthermore, there were main effects of trial type, *F*_(1,28)_ = 14.335, *p* = 0.001, $\eta_{\text{p}}^{2}$ = 0.339 and the amplitude decreased in error trials. Additionally, there were main effects of electrode, *F*_(1,28)_ = 9.807, *p* = 0.004, $\eta_{\text{p}}^{2}$ = 0.259. The Fz electrode had a larger amplitude than the Cz electrode. However, no interaction effects were significant, including the interaction effect of state * trial type (*F*_(1,28)_ = 2.505, *p* = 0.125, $\eta_{\text{p}}^{2}$ = 0.082), the interaction effect of state * electrode (*F*_(1,28)_ = 1.606, *p* = 0.215, $\eta_{\text{p}}^{2}$ = 0.054), the interaction effect of trial type * electrode (*F*_(1,28)_ = 0.968, *p* = 0.334, $\eta_{\text{p}}^{2}$ = 0.33) and the interaction effect of state * trial type * electrode (*F*_(1,28)_ = 0.036, *p* = 0.852, $\eta_{\text{p}}^{2}$ = 0.001).

For P2 latency, there was a main effect of state *F*_(1,28)_ = 8.106, *p* = 0.008, $\eta_{\text{p}}^{2}$ = 0.225, and the latency was longer in fatigue. However, there were no main effects of trial type *F*_(1,28)_ = 1.968, *p* = 0.172, $\eta_{\text{p}}^{2}$ = 0.066 and no interaction effects of state * trial type *F*_(1,28)_ = 2.705, *p* = 0.111, $\eta_{\text{p}}^{2}$ = 0.088 were significant.

For N2 amplitude, there was no main effect of state *F*_(1,28)_ = 0.339, *p* = 0.565, $\eta_{\text{p}}^{2}$ = 0.012. The amplitude increased significantly for error trials *F*_(1,28)_ = 18.68, *p* < 0.001, $\eta_{\text{p}}^{2}$ = 0.400. There was no main effect of electrode *F*_(1.127,31.549)_ = 0.335, *p* = 0.593, $\eta_{\text{p}}^{2}$ = 0.012. Moreover, no interaction effects were significant, including interaction of state * trial type (*F*_(1,28)_ = 1.164, *p* = 0.29, $\eta_{\text{p}}^{2}$ = 0.040), interaction of state * electrode (*F*_(1.142,31.963)_ = 1.288, *p* = 0.271, $\eta_{\text{p}}^{2}$ = 0.044), interaction of trial * electrode (*F*_(1.159,32.447)_ = 1.177, *p* = 0.295, $\eta_{\text{p}}^{2}$ = 0.040) and interaction of state * trial type * electrode (*F*_(1.134,31.75)_ = 0.0.13, *p* = 0.933, $\eta_{\text{p}}^{2}$ < 0.001).

The N2 latency was longer in the fatigue state *F*_(1,28)_ = 7.68, *p* = 0.01, $\eta_{\text{p}}^{2}$ = 0.215, but there was no difference between trial type *F*_(1,28)_ = 1.484, *p* = 0.233, $\eta_{\text{p}}^{2}$ = 0.050. Moreover, there was no interaction effect of state * trial type *F*_(1,28)_ = 0.973, *p* = 0.332, $\eta_{\text{p}}^{2}$ = 0.034 were significant.

The fatigue state decreased the P3 amplitude significantly *F*_(1,28)_ = 7.295, *p* = 0.012, $\eta_{\text{p}}^{2}$ = 0.207. Furthermore, the amplitudes decreased significantly in error trials, *F*_(1,28)_ = 23.599, *p* < 0.001, $\eta_{\text{p}}^{2}$ = 0.457 and there was a main effect of electrode (*F*_(1.307,36.609)_ = 9.527, *p* = 0.002, $\eta_{\text{p}}^{2}$ = 0.54), where the Pz electrode displayed the largest amplitude. However, no interaction effects were significant, including interaction of state * trial type (*F*_(1,28)_ = 0.694, *p* = 0.412, $\eta_{\text{p}}^{2}$ = 0.024), interaction of state * electrode (*F*_(1.305,36.527)_ = 0.51, *p* = 0.527, $\eta_{\text{p}}^{2}$ = 0.018), interaction of trial * electrode (*F*_(1.297,36.33)_ = 3.152, *p* = 0.074, $\eta_{\text{p}}^{2}$ = 0.101), and interaction of state * trial type * electrode (*F*_(1.266,35.434)_ = 0.391, *p* = 0.585, $\eta_{\text{p}}^{2}$ = 0.014).

There was a marginal main effect of state on P3 latency *F*_(1,28)_ = 3.689, *p* = 0.065, $\eta_{\text{p}}^{2}$ = 0.116, with a longer latency observed in the fatigue state. However, there was no main effects of trial *F*_(1,28)_ = 1.082, *p* = 0307, $\eta_{\text{p}}^{2}$ = 0.037 and no interaction effect of state * trial type (*F*_(1,28)_ = 0.412, *p* = 0.526, $\eta_{\text{p}}^{2}$ = 0.014).

## Discussion

The aim of this study was to identify which cognitive processes were impaired prior to error response under mental fatigue. The hypotheses were partially verified by our results. The behavioral data revealed more errors and shorter RTs after long-lasting task. Alongside the behavior data, the ERPs data revealed reduced P3 and P2 amplitudes and longer latency of P2 and N2 in the last period. All the results showed that the long-lasting task-induced fatigue. In error trials, as expected, P3 and P2 amplitudes were decreased, yet N2 amplitude was increased.

### Impaired Cognitive Processes in Mental Fatigue

Shorter RT indicates that the participants use a more rapid reaction strategy in mental fatigue state (Boksem et al., 2005, 2006). Moreover, our rating results showed that fatigue and negative emotion rose with time. Thus, the subjects were inclined to finish the task as quickly as possible and thus they may shifted to a new response strategy that conserved cognitive resources (Boksem et al., 2006). As a result, the processing was insufficient for responses, further shortening the RT and inducing more errors after the long-lasting task. The behavioral results indicated that the cognitive resources involved in error monitoring and behavior adjustment were reduced in the fatigue state.

The ERPs’ results were consistent with the behavioral results. Diminished P2 and P3 amplitudes and longer latency of P2 and N2 implied that memory updating, cognitive control, and attention were impaired in the fatigue. It is believed that diminished P3 is associated with deficit in sustained attention and memory updating (Watter et al., 2001; Chen et al., 2008), and is associated with resource reallocation (Falkenstein et al., 1994; Polich and Heine, 1996; Polich, 2007; Schapkin and Freude, 2013). Furthermore, attention and memory updating were inhibited, revealed by diminished P2 amplitude and longer latency of P2 and N2. These changes implied that fewer cognitive resources were available for memory updating (Taylor et al., 1990; Lefebvre et al., 2005; Lenartowicz et al., 2010; Ora et al., 2015), response selection and error monitoring (Patel and Azzam, 2005; Azizian et al., 2006; Gajewski et al., 2008; Folstein et al., 2008). Longer N2 latency in our study was consistent with findings in the literature (Schapkin and Freude, 2014). The results reported by Schapkin and Freude indicated that more cognitive resources were reallocated to the early stages of information processing, while the response selection lacked sufficient resources and processing time. Here, our results suggest that mental fatigue also reallocates more cognitive resources to early information processing and impairs response selection.

Our ERPs’ results were also consistent with previous works suggesting that the target-driven attention system, but not the stimulus-driven attention system, was easily affected in fatigue (Boksem et al., 2005, 2006). These studies found larger N2 latency and smaller P2 and P3 amplitudes in fatigue. Similar results have also been reported in the literature (Bruder et al., 1998; Guillem et al., 2006; Sumich et al., 2014) regarding impairment in active control and target-driven attention in patients with depression (Bruder et al., 1998) and schizophrenia (Guillem et al., 2006). These results found that more effort and attention were needed to finish a task. It induced hyper-activation in the medial temporal region, increasing the N1 and N2 amplitudes and decreasing the P2 and P3 amplitudes. Consistent with previous findings, our results showed that P2 and P3 amplitudes decreased and their latencies were longer in the fatigue state. It means that the active control and target-driven attention were impaired.

In summary, the target-driven attention system was affected and more cognitive resources were reallocated to the early information process which increased N2 and P2 latency, as well as decreased the P2 and P3 amplitudes. All the results implied that attention, memory updating and cognitive control were impaired in fatigue. And thus, the participants shifted the response selection strategy. Taken together, all these changes contributed to a higher error rate in the fatigue state.

### Neural Patterns Prior to Error Response

Smaller P3 and P2 amplitudes but larger N2 amplitude were observed in error trials in both the normal and fatigue states. These changes indicated that attention, memory updating, and cognitive control were impaired simultaneously in error responses. To our knowledge, it is the first report on the impairment of multiple cognitive processes before the error occurred under fatigue.

Diminished P2 amplitude and longer latency implied that there were faults in the onset of memory updating. P2 is related to the onset of memory updating (Taylor et al., 1990; Lefebvre et al., 2005; Lenartowicz et al., 2010), reflecting the evaluation of the inner representative or expectancy process including the identification and encoding processes (Luck and Hillyard, 1994; Watter et al., 2001; Ridderinkhof et al., 2003; Potts, 2004; Chen et al., 2008; Wessel et al., 2012; Schapkin and Freude, 2014; Shou et al., 2015). The onset of memory updating is the crucial step for successful performance (Ora et al., 2015). In the 2-back WM task, the new stimulus should be identified, encoded and compared with the memory block to evaluate whether it is the target (Watter et al., 2001; Chen et al., 2008). Our results revealed smaller P2 in error trials, suggesting that there were some faults in the onset of memory updating that lead to the error response.

Decreased P3 amplitude indicated that attention and memory updating were impaired prior to error response. P3 amplitudes decreased significantly in error trials, which showed that cognitive resources were reallocated to other processes and attention was impaired. Previous studies (Gajewski and Falkenstein, 2014) have found that cognitive resources were reallocated and impaired attention decreased the P3 amplitude during the 2-back WM task (Polich and Heine, 1996; Polich, 2007; Daffner et al., 2011b; Wild-Wall et al., 2011; Saliasi et al., 2013; Schapkin and Freude, 2013). P3 amplitude decreases in both Fz and Pz indicated that attention was impaired when errors are going to occur (Cael et al., 1974; Wilkinson and Seales, 1978; Sutton et al., 1982; Schapkin and Freude, 2014). Additionally, decreased P3 amplitude in error trials reflects a deficit in memory updating. Memory updating includes encoding, operation, search and selection of information. This stage is crucial for successful performance in a WM task. It is believed that P3 amplitude is an effective measurement of memory updating (Cael et al., 1974; Wilkinson and Seales, 1978; Sutton et al., 1982; Watter et al., 2001; Chen et al., 2008). Memory updating increased with better performance and led to increases in P3 amplitudes in Pz when subjects received n-back training (Cael et al., 1974; Wilkinson and Seales, 1978; Sutton et al., 1982; Zhao et al., 2013). In contrast, reduced P3 amplitudes during error trials indicated that memory updating was impaired and produced error responses.

Larger N2 amplitudes in error trials implied that decreased cognitive control leading to errors (Boksem et al., 2005, 2006; Caseras et al., 2006). It has been reported that N2 is related to cognitive control, including such processes as response selection and conflict detection in memory (Daffner et al., 2011b; Gajewski and Falkenstein, 2014). Some studies suggested that diminished P3 amplitude in WM tasks occurred when more resources were recruited in the cognitive control process, as indicated by increased N2 amplitude (Donkers et al., 2005; Jonathan Folstein et al., 2008; Daffner et al., 2011b; O’Connell et al., 2012). Increased N2 amplitude meant that more neural resources related to cognitive control were activated (Bruder et al., 1998, 2001; Daurignac et al., 2006; Guillem et al., 2006; Shu et al., 2014; Sumich et al., 2014; Pinal et al., 2015b; Zuj et al., 2017), it refers to hyper-activation in the medial temporal region, and since more resources were recruited for the same task but induced error responses, thus the neural efficiency was deceased based on the energy cost. All these results clearly indicate that decreased cognitive control is related to the error.

## Conclusion

In this study, we have demonstrated that simultaneous impairment of several cognitive processes under fatigue state may be the source of error responses in a WM task. Moreover, mental fatigue affected attention, impaired cognitive control and impaired memory updating, which induced more errors compared to the normal state. The behavioral data and ERPs results together verified that impairment of multiple cognitive processes contributes to an error in a single task.

## Data Availability Statement

The raw data supporting the conclusions of this article will be made available by the authors, without undue reservation, to any qualified researcher.

## Ethics Statement

The studies involving human participants were reviewed and approved by the China Astronaut Research and Training Center Committee. The patients/participants provided their written informed consent to participate in this study.

## Author Contributions

YX, JW, JL, WT, FM, CS YY, WZ, LW, HY, FX, and SC conceptualized and designed the study. The research idea was proposed by SC. Data collection and preliminary analysis were handled by JW and JL. Drafting and writing of the manuscript were handled by YX. YX contributed to the review and revised the manuscript. All authors have read and approved the final manuscript, and agreed to be accountable for the accuracy and integrity of this study.

## Conflict of Interest

The authors declare that the research was conducted in the absence of any commercial or financial relationships that could be construed as a potential conflict of interest.
